# Anti‐inflammatory activity of cyanidin‐3‐O‐glucoside and cyanidin‐3‐O‐glucoside liposomes in THP‐1 macrophages

**DOI:** 10.1002/fsn3.2554

**Published:** 2021-10-29

**Authors:** Xuefang Hao, Rongfa Guan, Haizhi Huang, Kai Yang, Lina Wang, Yuanfeng Wu

**Affiliations:** ^1^ Zhejiang Provincial Key Laboratory of Biometrology and Inspection and Quarantine China Jiliang University Hangzhou China; ^2^ College of Food Science and Technology Zhejiang University of Technology Hangzhou China; ^3^ School of Biological and Chemical Engineering Zhejiang University of Science and Technology Hangzhou China

**Keywords:** anti‐inflammatory activity, apoptosis, cyanidin‐3‐O‐glucoside, liposome, THP‐1 Macrophage

## Abstract

Cyanidin‐3‐O‐glucoside (C3G) is a kind of water‐soluble pigment widely existing in many plants. It has strong antioxidant and anti‐inflammatory activities. However, C3G cannot exist stably for a long time because of the phenolic hydroxyl groups in its structure. Liposome technology could improve the stability and bioavailability of compounds. Based on our previous studies, C3G liposomes prepared by ethanol injection method have a certain stability in two weeks of storage. In this study, THP‐1 macrophages treated with C3G and C3G liposomes can reduce the levels of inflammatory‐related factors, such as tumor necrosis factor‐a (TNF‐a), interleukin (IL)‐1β, IL‐6, and IL‐8, stimulated by lipopolysaccharide (LPS). Further studies showed that the LPS induction could increase the level of phosphorylated nuclear transcription factor NF‐κB and phosphorylated IkBa, while C3G and C3G liposomes could inhibit the expression of phosphorylated proteins. Moreover, C3G and C3G liposomes could protect macrophages from apoptosis. In conclusion, C3G prepared by liposome technology exhibits anti‐inflammatory activity, which provides a theoretical basis for the food industry to study functional food.

## Graphical abstract

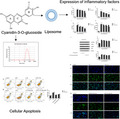

## 1 INTRODUCTION

Inflammation is a response of the body to stimulation, which may be beneficial or harmful. Inflammation may either be acute or chronic, and is related to the homeostasis of physiological systems in vivo. Inflammation is associated with many diseases and is the basis of most pathological processes. The main causes of inflammation are infection and tissue damage, which can lead to the concentration of leukocytes and plasma proteins (Medzhitov, 2008). Chronic inflammation is involved in many diseases, including diabetes, obesity, atherosclerosis, and cancer (Coussens & Werb, 2002; Grivennikov et al., 2010; Hotamisligil, 2006; Libby et al., 2002). THP‐1 macrophages model is usually used to study the anti‐inflammatory effects of compounds, and THP‐1 cells differentiate well when they are exposed to 25‐nm phorbol 12‐myristate 13‐acetate (PMA) for 48 h and then cultured with fresh medium for 24 h (Daigneault et al., 2010; Lund et al., 2016). LPS could be used to induce macrophages to release proinflammatory mediators directed by the TLR4‐mediated MyD88‐dependent pathway, and in the MyD88‐dependent pathway, MyD88 activated the NF‐κB pathway (Amini et al., 2018; Lu et al., 2008). Studies have shown that exposure of cells to LPS or inflammatory factors, such as tumor necrosis factor‐a (TNF‐a), interleukin (IL)‐1β, ultraviolet radiation or other stimuli, could activate NF‐κB (Baldwin, 1996). Macrophages secreted inflammatory factors, such as TNF‐a, IL‐1β, and IL‐6, which are controlled by the NF‐κB signal transduction pathway (Grivennikov & Karin, 2010).

Anthocyanins are a kind of natural plant pigments, which are the cause of blue, purple, and red color in many plants, and they mainly exist in the form of glycosides. Anthocyanins are widely found in fruits and vegetables and mainly absorbed in the stomach and small intestine. Anthocyanins have antioxidant, anti‐inflammatory, anticancer, and other biological activities (He & Giusti, 2010; Prior, 2003). Common berries, including blackberry, blueberry, cranberry, raspberry, and strawberry, are all rich in anthocyanins, and their extracts could inhibit the growth of human cancer cells and stimulate apoptosis (Seeram et al., 2006). Anthocyanins widely exist as biologically active ingredients and are relatively easy to obtain. They are widely used in various cell models in vitro because of their natural sources and unique biological activities. Cyanidin‐3‐O‐glucoside (C3G) is one of the most widespread anthocyanins in fruits and vegetables, and vitro studies have shown that C3G could inhibit the inflammatory pathway regulated by NF‐κB in adipocytes (Molonia et al., 2020). Anthocyanins are flavonoids whose stability is affected by many factors, such as temperature, pH, light, and metal ions (West & Mauer, 2013). Considering the potential application value of anthocyanins, improving their stability and bioavailability is necessary (Braga et al., 2018).

Liposomes, which are similar to cell membranes in structure, are often used as drug delivery systems. Liposomes are considered safe nanocarriers because of their biocompatibility, biodegradability, chemical stability, and low toxicity (Bozzuto & Molinari, 2015). Moreover, nanodrugs could ameliorate drug dissolution, and address half‐life problems, further to improve the drug absorption rate and reduce the renal clearance rate (Li & Huang, 2008). At present, the development of liposome technology has made some progress. Evidence indicates that anthocyanin liposomes prepared by pH gradient loading method have significantly improved stability, antioxidant activity, and skin permeability and could be stable for 14 days under physiological conditions in vitro (Lee & Na, 2019). Some researchers have found that the liposomes prepared by extract of black carrots are still maintained stable after 21 days of storage (Guldiken et al., 2018). Many studies have shown that C3G‐loaded nanoparticles have good encapsulation and loading efficiency and can further improve the stability of C3G, which provides a basis for the study of the application of C3G nanoparticles in functional food.

There are many studies on the anti‐inflammatory activity of C3G, but few have studied the anti‐inflammatory activity of C3G liposomes in macrophages. In this work, the effects of C3G and C3G liposomes on the levels of cytokines, such as TNF‐a, IL‐1β, IL‐6, and IL‐8 in the inflammatory cell model were studied and the expression of inflammatory‐related proteins were analyzed to further explore their anti‐inflammatory mechanisms. In addition, we also studied the effects of C3G and C3G liposomes on cell apoptosis in LPS‐stimulated THP‐1 macrophages.

## 2 MATERIALS AND METHODS

### 2.1 Reagents

C3G was purchased from Chengdu Biopurify Phytochemicals Ltd. Phorbol 12‐myristate 13‐acetate (PMA) and lipopolysaccharide (LPS) (*Escherichia coli*, serotype 0,127: B8) were purchased from Sigma Chemicals Co. (Sigma‐Aldrich). Cell counting kit‐8 (CCK‐8) was obtained from Shanghai Beyotime Biotechnology Co., Ltd. Cell culture reagents, as well as RPMI‐1640 medium, were purchased from Hyclone Laboratories (Hyclone Laboratories, Inc.), and fetal bovine serum (FBS), phosphate‐buffered saline (PBS) were acquired from Beijing Solarbio Life Sciences Ltd. NF‐κB p65 rabbit monoclonal antibody (p65), phospho‐NF‐κB p65 (p‐p65), IκB alpha rabbit monoclonal antibody (IκBa), phospho‐IκBa rabbit monoclonal antibody (p‐IκBa), β‐actin rabbit monoclonal antibody, and secondary antibodies were obtained from Cell Signaling Technology, Inc. Human cytokine enzyme‐linked immunosorbent assay (ELISA) kits were from Hangzhou Lianke Technology. All other chemicals were of reagent grade.

### 2.2 Preparation of C3G liposomes

C3G liposomes were prepared using the ethanol injection method with some modification (Quan et al., 2020). Appropriate amounts of cholesterol and lecithin were added into the right amount of absolute ethanol in the proportion of 1:3, and the mixture was dissolved completely by ultrasonication. Then, 3 mg of C3G were added, and they were dissolved completely. The obtained solution was slowly injected into 10 ml of deionized water and stirred for a period of time. Thereafter, the film was evaporated at 55℃ and 60 rpm until a uniform film was formed. Finally, an appropriate amount of deionized water was added to hydrate the liposomes and the prepared liposomes were filtered through a 0.22 μm filter for further use.

### 2.3 Characteristics of C3G liposomes

The particle size of prepared liposomes was determined by a particle size analyzer (Zetasizer NanoZS 90, Malvern Company). The encapsulation efficiency (%) of C3G liposomes was measured by previous study to quantify the concentration of encapsulated C3G in the aqueous phase (Gorjian et al., 2021). A 2‐ml aliquot of the prepared liposome solution was collected and centrifuged by using a freeze centrifuge (Beckman) at 7104 g for 30 min at 4°C, and the total amount of C3G absorbance of each sample was recorded at 540 nm by a UV spectrophotometry(UV‐1200, China). Finally, the encapsulation efficiency (EE %) was calculated by Equation (1).

(1) $$\text{EE}\%=\frac{W_{\text{en}}}{W_{\text{total}}}100\%$$

Where, *W*_total_ and *W*_en_ are the amount of free C3G, and the total amount of C3G present in liposomes, respectively. These variables were measured with a spectrophotometer and calculated afterward.

### 2.4 Cell culture and establishment of inflammation model

Human THP‐1 monocyte cell line was purchased from Shanghai Gaining Biotechnology Co., Ltd. Cells were cultured in RPMI‐1640 medium supplemented with 10% (v/v) FBS, penicillin (100 kU/L), and streptomycin (100 g/L) at 37°C in a humidified atmosphere of 5% CO_2_. THP‐1 cells were incubated with PMA (40 ng/ml) for 48 h to differentiate into macrophages and were treated with fresh medium for 24 h prior to use.

### 2.5 Cell viability assay

Cell viability was determined using a CCK‐8 assay kit. The cells within logarithmic growth phase were seeded in 96‐well plates (5 × 10^4^ cells/ml), incubated with different concentrations of C3G or C3G liposomes for 24 h, and added with CCK‐8 solution (10 μl) for 4 h at 37°C. The optical density (OD) was measured with a microplate spectrophotometer reader (iMark, Bio‐Rad) at λ = 450 nm.

### 2.6 Analysis of cytokine production

Inflammatory factors, including TNF‐a, IL‐1β, IL‐6, and IL‐8 were detected using specific ELISA kits.THP‐1 cells were incubated with PMA for 48 h in 96‐well plates and changed with fresh medium for 24 h. The cells were treated with LPS (1 μg/ml) for 24 h after incubating with C3G or C3G liposomes for 12 h. The cell culture supernatants were collected and analyzed according to the manufacturer's instructions.

### 2.7 Western blot analysis

The intracellular levels of IκB‐α, p‐IκB‐α, p65 and p‐p65 were assessed by Western blot analysis. THP‐1 cells were seeded (1 × 10^6^ cells/ml) in 6‐well plates for 48 h to differentiate into macrophages and treated with fresh medium for 24 h. The macrophages were incubated with LPS (1 μg/ml) for 6 h after incubating with compounds for 12 h. Subsequently, the treated cells were washed with PBS and then lysed using lysis buffer on ice for 10 min. The lysates were centrifuged at 21,756 g for 15 min at 4°C, and supernatants were collected and stored at −80°C. Bradford method was used to determine the concentration of the extracted protein. The equal quantities of protein samples were separated via sodium dodecyl sulfate polyacrylamide gel electrophoresis and then transferred to polyvinylidene difluoride membranes. The membranes were blocked using 5% bovine serum albumin in tris‐buffered saline tween (TBST) at room temperature and incubated with the first primary antibodies overnight at 4℃. The membranes were washed with TBST buffer three times before they were incubated with the second antibodies for 1 h at room temperature. Thereafter, the membranes were washed three times with TBST for 20 min every time. Finally, immunodetection was developed using an ECL reagent and scanned with a chemiluminescence imaging system (Tanon).

### 2.8 Immunofluorescence assays

The nuclear translocation of the NF‐κB p65 subunit was observed using immunofluorescence assay. THP‐1 cells were seeded onto 6‐well plates with pretreated cell climbing plates. After the cells differentiated into macrophages, they were pretreated with C3G or C3G liposomes for 12 h, followed by stimulation with LPS for 24 h. Then, the cells were washed three times with PBS and fixed with glutaraldehyde aqueous and permeabilized with 0.5% Triton X‐100. Subsequently, the cells were blocked at room temperature and incubated with antibody NF‐κB p65 overnight. After the cells were washed, they were incubated with goat antirabbit IgG‐FITC for 1 h. Nuclei were stained with DAPI, and the coverslips were mounted on slides with an anti‐fluorescence quenching agent. A fluorescence microscope (Nikon, TE‐Eclipse 300) was used to observe and capture fluorescence images.

### 2.9 Transmission electron microscope (TEM) for cell morphology observation

The cells within logarithmic growth phase were seeded onto 6‐well plates (1 × 10^6^ cells/ml) with C3G or C3G liposomes for 12 h. The collected cells were washed twice with precooled PBS, and fixed overnight at 4℃ in 2.5% glutaraldehyde solution. The samples were prepared through the basic method of sample pretreatment of TEM, and the cell images were observed by a transmission electron microscope (JEM‐2100, Japanese Electronics Co., Ltd.).

### 2.10 Apoptosis experiments

The collected cells were washed twice with precooled PBS, and resuspended with 1× binding buffer to determine the effects of C3G or C3G liposomes in the presence or absence of LPS on apoptosis. Briefly, 5 μl of annexin‐v‐FITC and 10 μl of propidium iodide were added into each tube of cells. After the cells were mixed gently by using a vortex mixer, they were incubated for 5 min in the dark at room temperature. Finally, a flow cytometry (BD Accuri™ C6)was performed.

### 2.11 Statistical analysis

GraphPad Prism Software was used for statistical analysis, and the results are presented as means ± SD, as specified in the figure legends. The significance of differences was calculated by one‐way ANOVA and value of *p* < .05 was considered statistically significant, and specific significance values are stated in the figure legends.

## 3 RESULTS AND DISCUSSION

### 3.1 Liposomes characterization

**FIGURE 1:**
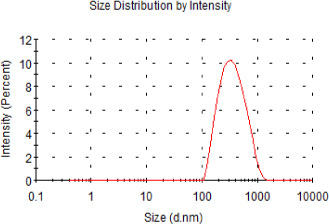
The size distribution of the prepared C3G liposomes

As indicated in Figure 1, the liposomes presented a normalized size distribution curve, and the main particle size of C3G liposome ranged from 100 nm to 1000 nm, and the EE (%) of C3G liposome reached 70%. Based on our previous studies, the C3G liposomes prepared by ethanol injection method showed a stable particle size of about 258.9 ± 5.06 nm and an encapsulation efficiency of 77.5%, and the C3G liposomes had a certain stability during the storage period of two weeks (Liang et al., 2019). It was reported that anthocyanins cannot stay in the upper digestive tract for a long time, which may lead to a decrease in their biological activity (Tarone et al., 2020). However, liposome technology could protect anthocyanins from the external environment and slow down the release of anthocyanins in simulated gastric juice (Zhao et al., 2017). The encapsulation of liposomes could enhance the stability and the cellular uptake, as well as the antioxidant activity of anthocyanins in vitro (Sun et al., 2021). Good physicochemical characterizations of C3G liposomes were observed, which prepared by ethanol injection method, and this method was simple and safe. These results expect to provide theoretical guidance for future research on functional foods.

### 3.2 Effects of C3G and C3G liposomes on cell viability

**FIGURE 2:**
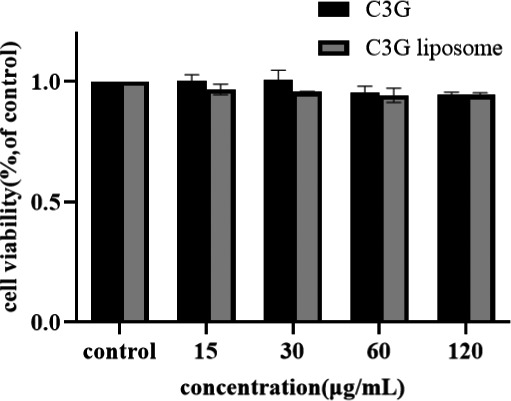
THP‐1 macrophages were incubated with different concentrations of C3G or C3G liposomes for 24 h. Cell viability was assessed by CCK‐8 assay

In the cell viability test, THP‐1 cells were incubated with or without C3G or C3G liposomes for 24 h. Figure 2 shows that the difference in cell activity between C3G and liposome groups compared with the control groups was not significant, and cell viability was >90%. Thus, C3G and C3G liposomes were not toxic to THP‐1 macrophages at the amount ranges examined and could be used in subsequent experiments.

### 3.3 Effects of C3G and C3G liposomes on the production of TNF‐a, IL‐1β, IL‐6, and IL‐8 in LPS‐induced THP‐1 macrophages

**FIGURE 3:**
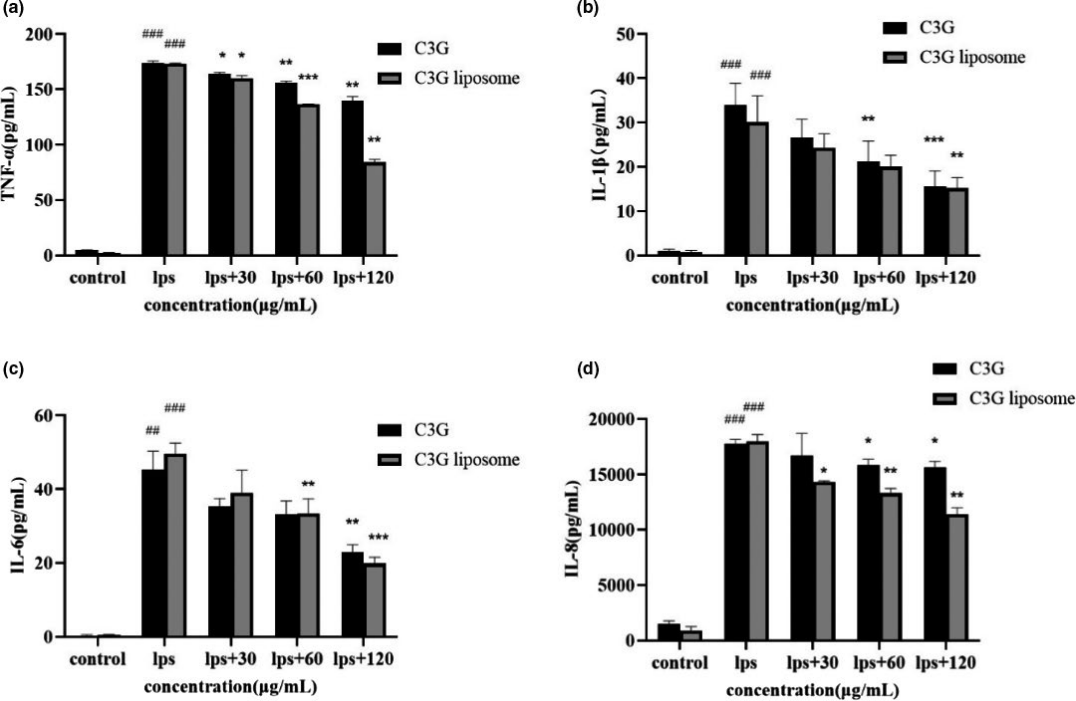
Effects of C3G or C3G liposomes on TNF‐α, IL‐1β, IL‐6, and IL‐8 levels in LPS‐treated THP‐1 macrophages. Cells were treated with indicated doses of C3G or C3G liposomes for 12 h and then stimulated with or without LPS (1 μg/ml) for 24 h. Results are the mean ± SD of triplicate determinations. ^##^ *p* < .01, ^###^ *p* < .001 compare with the control group. *p < .05, **p < .01, ***p < .001 compare with the LPS group

TNF‐a, IL‐1β, IL‐6, and IL‐8 are important inflammatory factors in the process of inflammation. In LPS‐stimulated THP‐1 macrophages, C3G and C3G liposomes inhibited the production of inflammatory factors, such as TNF‐a, IL‐1β, IL‐6, and IL‐8. Figure 3 shows that LPS significantly increased the secretion of inflammatory factors (*p* < .0001). Thus, LPS significantly increased the production of proinflammatory cytokines, while cytokines production decreased in a dose‐dependent manner with C3G and C3G liposomes treatment.

In order to compare the effects of C3G and C3G liposomes on the release of inflammatory factors, we added C3G and C3G liposomes with the same concentration under the same conditions. As shown in Figure 3a, when the drug concentrations were 60 and 120 μg/ml, the release of TNF‐a in C3G groups were 155.59 ± 1.94 and 140.00 ± 3.48 pg/ml, respectively, while that in liposome groups were 136.77 ± 0.084 and 84.29 ± 2.70 pg/ml, respectively. Similarly, when exploring the expression of IL‐8, the decrease rate of inflammatory factors expression in liposome groups was more obvious after adding drugs. When the concentration of C3G liposome was 120 μg/ml, IL‐8 release was decreased by 36.68% ± 1.25% compared with the LPS group (*p* < .001), while that in C3G groups was 11.96% (*p* < .05). These results may be related to the stability and sustained release effect of liposomes.

Figure 3(b,c) show that C3G and C3G liposomes groups reduced IL‐1β and IL‐6 production more significantly in a dose‐dependent manner compared with the LPS group. And evidence had indicated that polyethylene glycol‐coated gold anthocyanin nanoparticles attenuate neuro‐inflammatory cytokines IL‐1β and reduce apoptosis more significantly compared with anthocyanin alone (Kim et al., 2017). However, our results showed that there was no significant difference in the release of IL‐6 and IL‐1β between C3G and C3G liposomes groups. According to reports, LPS is an important exogenous mediator that can induce the body's inflammatory response and is often used to establish inflammation models. Anthocyanins have been confirmed to inhibit the expression of cytokines in vitro and in vivo (Jiang et al., 2020; Qiu et al., 2021; Zhang, Lang, et al., 2020; Zhang, Xu, Zhao, et al., 2020). In this study, we found that C3G and C3G liposomes ameliorated LPS‐induced proinflammatory cytokines expression.

### 3.4 Effects of C3G or C3G liposomes on the p‐p65 and p‐IκBα expression in LPS‐induced THP‐1 macrophages

**FIGURE 4:**
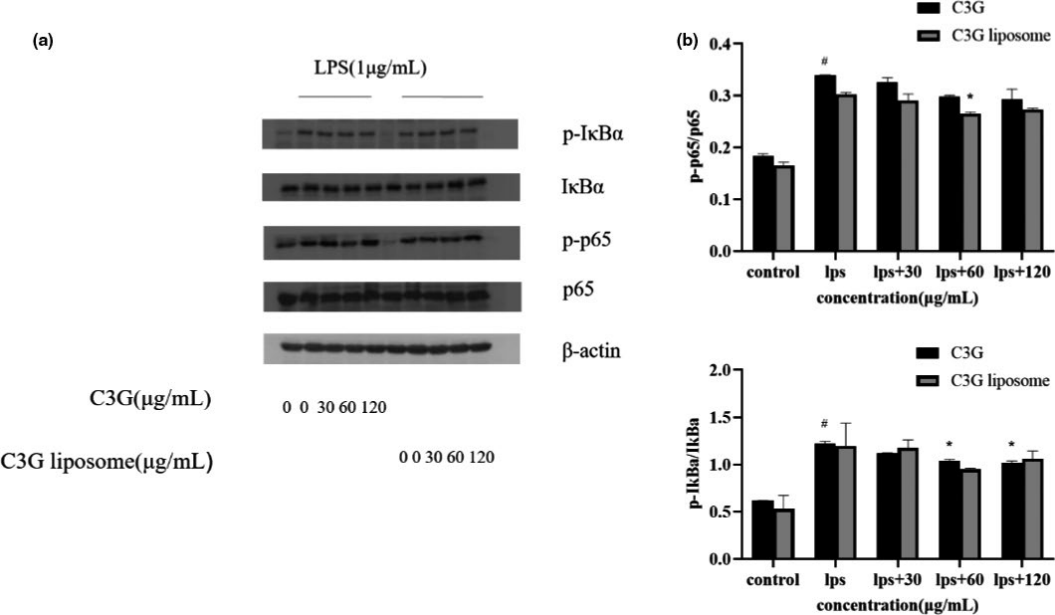
Inhibition of C3G or C3G liposomes on the protein levels of p65, p‐p65, IκBα, and p‐IκBα in THP‐1 macrophages stimulated by LPS. THP‐1 macrophages were treated with different concentrations of C3G or C3G liposomes for 12 h, and then treated with or without LPS (1 μg/ml) for 6 h. The protein expression of p65, p‐p65, IκBα, and p‐IκBα were measured by Western blot assay. Protein levels are corrected to β‐actin. All data are presented as mean ± SD of three independent experiments. ^##^ *p* < .01, ^###^ *p* < .001 compare with the control group. *p < .05, **p < .01, ***p < .001 compare with the LPS group

The expression of proinflammatory genes is affected by the NF‐κB signaling pathway. To determine whether the molecular mechanism of the anti‐inflammatory reaction of C3G or C3G liposomes is related to the NF‐κB signaling pathway, we used Western blot analysis to analyze the expression levels of p65, p‐p65, IκBα, and p‐IκBα proteins. As shown in Figure 4a, p‐p65 and p‐IκBα expression was induced by LPS. After treatment with C3G or C3G liposomes, the expression of phosphorylated proteins tended to decrease. Therefore, C3G or C3G liposomes may inhibit the activation of the NF‐κB pathway by inhibiting the phosphorylation proteins. The results showed that p‐p65 and p‐IκBα expression was inhibited by C3G or C3G liposomes within a certain concentration range, and the inhibition was enhanced with the increase in concentration.

**FIGURE 5:**
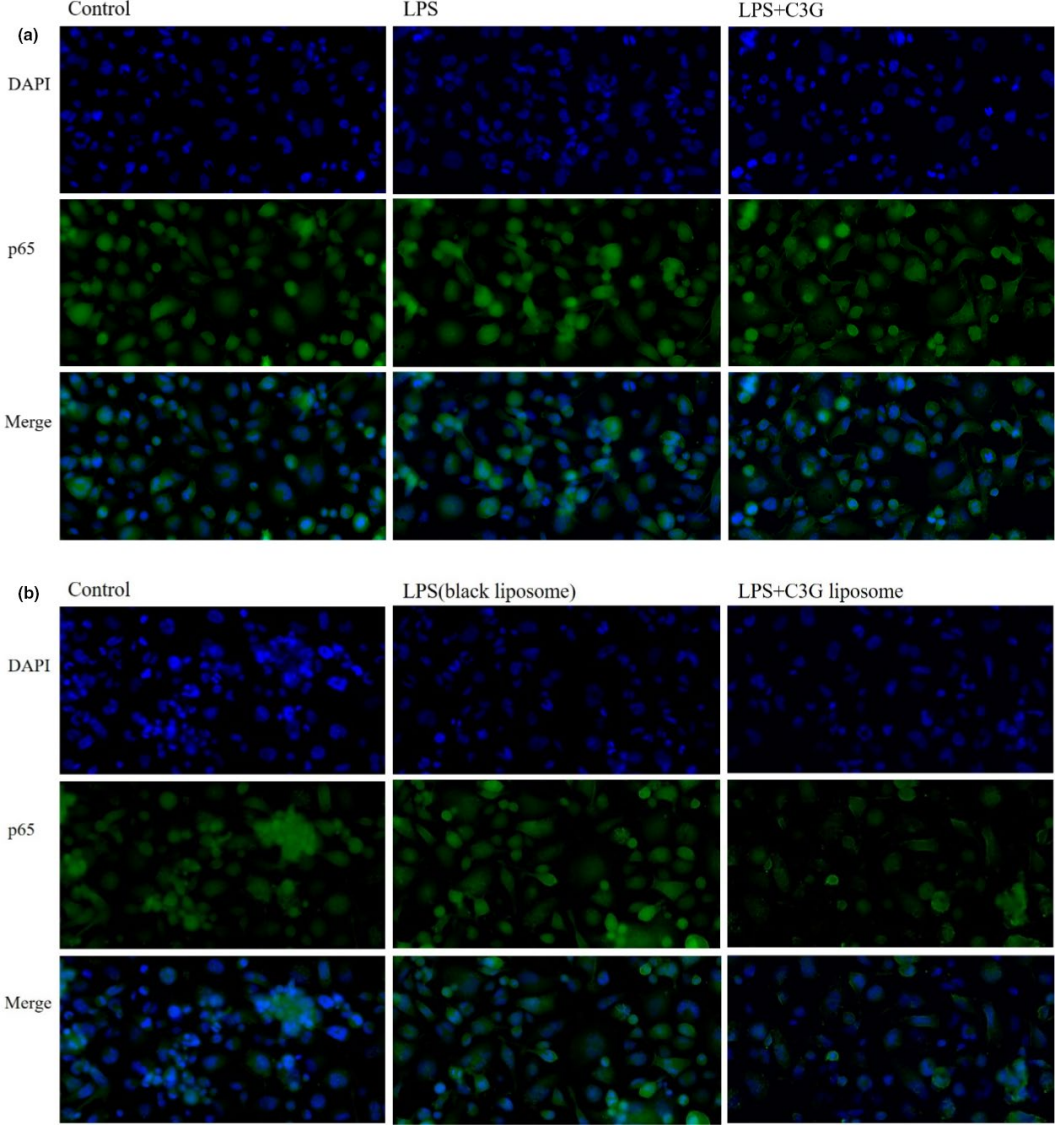
The localization of the p65 subunit of the NF‐κB complex with immunofluorescence staining. (a) THP‐1 macrophages were treated with C3G in the presence or absence of LPS; (b) THP‐1 macrophages were treated with C3G liposomes in the presence or absence of LPS

In addition, we detected NF‐κB nuclear translocation using immunofluorescence staining. As shown in Figure 5, when macrophages were exposed to LPS, the protein level of p65 was upregulated in the nucleus. However, C3G or C3G liposomes treatment dramatically inhibited p65 translocation.

The occurrence of inflammation is related to many transcription factors, which may involve a single signal‐transduction pathway or multiple pathways at the same time. Inflammation exists in the development of many diseases and is closely associated with the immune system. It is considered as a reaction of the body to tissue damage, usually accompanied by the release of various signal factors in the inflammatory area. The activation of NF‐κB p65 pathway has been identified as a major contributor of inflammation. As an endotoxin commonly found in the bacteria membrane of gram‐negative bacteria, LPS can cause the production of various oxidative stress and inflammation markers, and induce the synthesis and secretion of interleukins (Bhardwaj et al., 2021). When stimulated by LPS, IκBα is phosphorylated by its kinase IKK, and phosphorylation of IκBα further leads to its own ubiquitination and proteasome degradation, resulting in the activation of NF‐κB and its transport into the nucleus and inducing the production of inflammation‐related genes(Ren et al., 2020; Sun et al., 2020). NF‐κB and TNF‐a are the key signal factors in the inflammatory response. As shown in Figures 3a and 4, the expression of NF‐κB and TNF‐a increased significantly in the LPS‐treated group compared with the blank control group. The model of LPS‐stimulated macrophages has been widely used to study the mechanism of anti‐inflammatory drugs (Qian et al., 2021). Previous studies have reported that LPS induced the high expression of p‐p65, p‐IKKα/β, and p‐IκBα in lung tissue (Yang et al., 2021). Furthermore, evidence suggest that C3G from purple rice has been shown to significantly downregulate IkBa degradation and p‐p65 level by inhibiting the NF‐κB signaling pathway in IL‐1β‐stimulated human chondrocytes (Wongwichai et al., 2019). In these experiments, we observed that LPS stimulation leaded to NF‐κB activation confirmed by the increase in p‐IkBa content and p65 nuclear translocation through Western‐blot analysis and immunofluorescence assay. Furthermore, our study indicated that p‐IkBa increase and p65 translocation were remarkably reduced by C3G and C3G liposomes. Therefore, previous findings, together with our results (Figure 4) suggest that the NF‐κB signaling pathway might be involved in the suppressive effects of C3G or C3G liposomes on the release of proinflammatory cytokines in LPS‐induced THP‐1 macrophages.

NF‐κB activation could promote the release of proinflammatory cytokines. A previous study in metabolic syndrome subjects has reported that anthocyanins relieve inflammation by inhibiting the expression of NF‐κB‐dependent genes IL‐6 and TNF‐a (Aboonabi & Aboonabi, 2020). Also, Li et al. reported that the inhibition of the ROS/ NF‐κB pathway could decrease IL‐1β, IL‐6, and IL‐8 expression in macrophages (Li et al., 2020). The present study aimed to show the anti‐inflammatory effects of C3G and C3G liposomes through the inhibition of the NF‐κB signaling pathway by determining the expression of NF‐κB target genes. The results indicated that C3G and C3G liposomes inhibited the expression of NF‐κB‐associated genes TNF‐a, IL‐6, IL‐1β, and IL‐8.

### 3.5 TEM of THP‐1 macrophages

**FIGURE 6:**
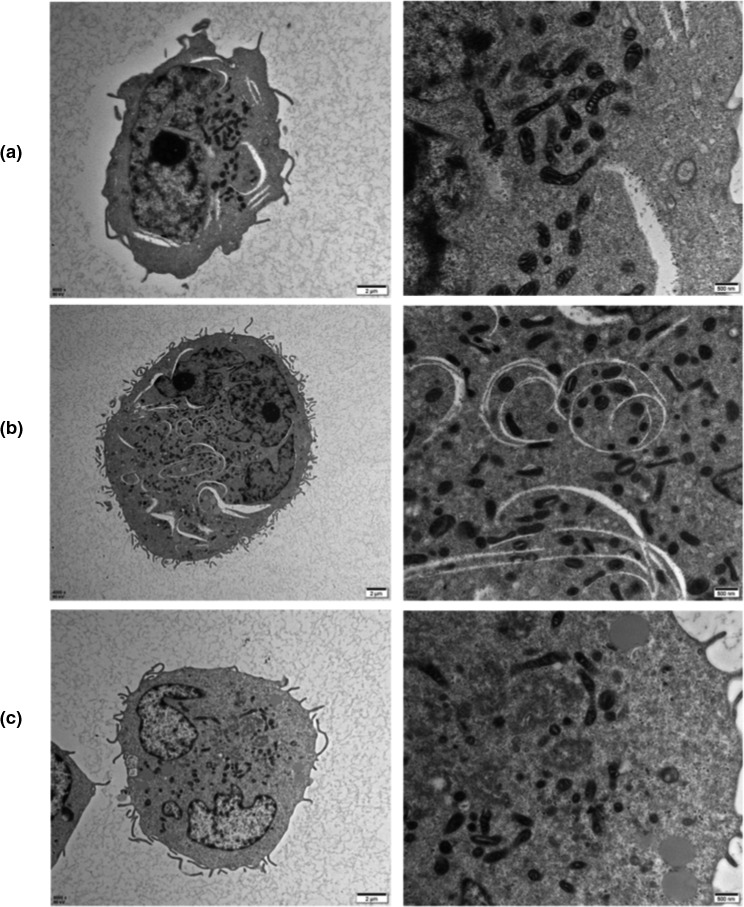
TEM images of cells treated with C3G or C3G liposomes for 12 h. (a) Control; (b)Simple1: C3 G;(c) Simple2: C3G liposome

The treated cells were observed under TEM to understand the effects of C3G or C3G liposomes on cell structure and mitochondria contents after co‐incubation with cells for a period of time, Figure 6 shows that the coincubation of C3G or C3G liposomes had a negligible effect on mitochondria contents in cells compared with the control group. C3G and C3G liposomes had no effect on cell structure, which corresponded to the results of the cell viability experiment.

Mitochondria are the main source of ATP and ROS, as well as a key organelle for maintaining cellular homeostasis. Mitochondrial dysfunction increase the production of ROS and further induce inflammation (Barrera et al., 2021). Mitochondria are the main target of flavonoids (Lagoa et al., 2011). Oxidative stress could cause mitochondrial dysfunction. Anthocyanins could alleviate mitochondrial dysfunction and promote the distribution of mitochondrial components (Parrado‐Fernandez et al., 2016). Some flavonoids have been proven to improve the decline of mitochondrial function, and researchers have found that the decline of mitochondrial function was related to a decrease in glutathione levels and lipid peroxidation (Franco et al., 2010). According to the experimental results, the incubation of C3G or C3G liposomes exerted negligible effects on cellular mitochondria, which provided theoretical support for subsequent experiments.

### 3.6 Results of the apoptosis experiment

**FIGURE 7:**
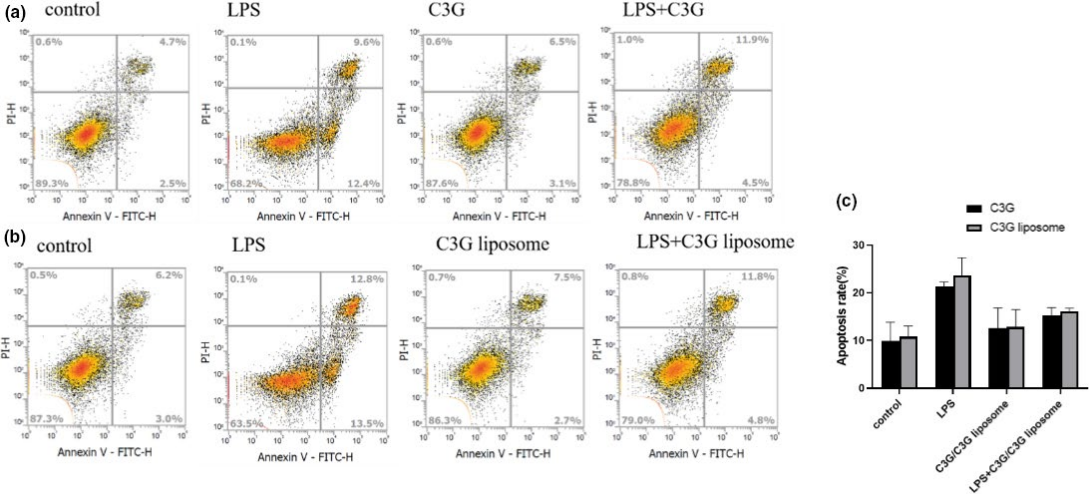
Apoptosis experiments were visualized by flow cytometry. (a) THP‐1 macrophages were incubated with C3G (120 μg/ml) in the presence or absence of LPS. (b) THP‐1 macrophages were incubated with C3G liposome (120 μg/ml) in the presence or absence of LPS. (c) Changes in the rate of cellular apoptosis

According to the results shown in the Figure 7, we found that the apoptosis rate of cells after LPS treatment was significantly increased compared with the control group. The apoptosis rate of C3G and C3G liposomes alone was not significantly different from that of the control group. However, compared with the LPS group, pretreatment with C3G or C3G liposomes before LPS treatment resulted in a decrease of 6.05% ± 0.45% or 7.55% ± 2.15% in cell apoptosis, respectively.

Apoptosis may be related to many factors and controlled by apoptosis‐related genes. Oxidative stress can cause apoptosis via various mechanisms. Mitochondrial dysfunction and endoplasmic reticulum stress induction may cause oxidative stress. Excessive ROS production results in oxidative stress, which can lead to lipid oxidation and apoptosis (Valko et al., 2007). In this study, we found that LPS could promote cellular apoptosis. When studying the effect of LPS on myocardial function in rats, the researchers found that LPS stimulation could cause swelling, fragmentation and reduction of cristae density in mitochondrial morphology, as well as a decrease in the activity of mitochondrial complex IV (Zhang, Xu, Zhu, et al., 2020). Based on previous research findings, C3G could effectively prevent ultraviolet‐induced apoptosis of HaCaT cells by scavenging ROS (He et al., 2017). Previous studies have confirmed that NF‐κB is also related to cellular apoptosis. Haihua Zhang found that Echinacea polysaccharide may reduce inflammation and cellular apoptosis by inhibiting the TLR4/NF‐κB signaling pathway, thereby slowing down LPS‐induced lung injury. However, some researchers have found that LPS could induce the activation of NF‐κB, but NF‐κB had no significant effect on LPS‐induced apoptosis (Zhang, Lang, et al., 2020; Zhu et al., 2020). Evidence shows that anthocyanin‐rich riceberry bran extract could prevent apoptotic liver damage partly by attenuating oxidative stress and suppressing the activation of the NF‐κB pathway (Arjinajarn et al., 2017). Anthocyanins prevented cell apoptosis, which may be mediated by multiple molecular mechanisms.

Studies have confirmed that TNF is an important mediator in the proinflammatory response, which not only participates in the induction of NF‐κB, but also induces apoptosis and regulates immunity (Fajgenbaum & June, 2020; Mei et al., 2021). As a key proinflammatory cytokine, TNF‐a might participate in the lymphocyte‐mediated adaptive immune response by initiating apoptosis (Li et al., 2021). C3G may inhibit apoptosis by suppressing the activation of cleaved caspase‐9/3 or p53 related protein and regulating the expression of apoptosis genes in hydrogen peroxide‐induced HepG2 cells (Tan et al., 2020). Phenolic compounds carvacrol could reduce LPS‐induced inflammation by inhibiting the transcriptional activation of NF‐κB in macrophages, and exhibited antiapoptotic activity (Somensi et al., 2019). However, some studies have found that flavonoids promote apoptosis and inhibit the growth of colon cancer cells by inducing ROS production (Dükel et al., 2021). Quercetin could promote mitochondria‐mediated apoptosis through NF‐κB inhibition in human cervical cancer cells (Vidya Priyadarsini et al., 2010). These results may be caused by the difference in the molecular mechanism of drugs in different cell models. Therefore, C3G liposomes have protective effects from LPS‐induced apoptosis, which may be partly related to the NF‐κB pathway, but the specific mechanism should be further studied.

## 4 CONCLUSIONS

In recent years, there have been many studies on the anti‐inflammatory activity of C3G, but there are few studies on the anti‐inflammatory activity of C3G liposomes. Our paper comparatively studied the anti‐inflammatory and antiapoptotic effects of C3G and C3G liposomes in vitro, and found that C3G liposomes have higher activity in inhibiting certain inflammatory factors. The encapsulation of liposomes has a certain protective effect on C3G. With the continuous maturity of liposome technology, the shortcomings of C3G that cannot exist stably in the natural environment for a long time are expected to be improved. Encapsulating C3G in liposomes to further study the functional activity of C3G has certain theoretical support and reference significance for future research on functional foods.

## CONFLICT OF INTEREST

The authors declare that they do not have any conflict of interest.

## AUTHOR CONTRIBUTIONS

**Xuefang Hao:** Data curation (lead); Writing‐original draft (lead). **Rongfa guan:** Data curation (lead); Funding acquisition (lead); Writing‐original draft (lead); Writing‐review & editing (lead). **haizhi huang:** Data curation (supporting); Writing‐original draft (supporting); Writing‐review & editing (lead). **Kai Yang:** Methodology (supporting). **ln wang:** Methodology (supporting). **yuanfeng wu:** Methodology (supporting).

## Data Availability

The data underlying this article will be shared on reasonable request to the corresponding author.
